# Association of preconception dysmenorrhea with obstetric complications: the Japan Environment and Children’s Study

**DOI:** 10.1186/s12884-021-04347-7

**Published:** 2022-02-15

**Authors:** Tsuyoshi Murata, Yuta Endo, Toma Fukuda, Hyo Kyozuka, Shun Yasuda, Akiko Yamaguchi, Akiko Sato, Yuka Ogata, Kosei Shinoki, Mitsuaki Hosoya, Seiji Yasumura, Koichi Hashimoto, Hidekazu Nishigori, Keiya Fujimori, The Japan Environment and Children’s Study (JECS) Group, Michihiro Kamijima, Shin Yamazaki, Yukihiro Ohya, Reiko Kishi, Nobuo Yaegashi, Koichi Hashimoto, Chisato Mori, Shuichi Ito, Zentaro Yamagata, Hidekuni Inadera, Takeo Nakayama, Hiroyasu Iso, Masayuki Shima, Youichi Kurozawa, Narufumi Suganuma, Koichi Kusuhara, Takahiko Katoh

**Affiliations:** 1 Fukushima Regional Center for the Japan Environment and Children’s Study, 1 Hikarigaoka, Fukushima, 960-1295 Japan; 2 Department of Obstetrics and Gynecology, Fukushima Medical University School of Medicine, 1 Hikarigaoka, Fukushima, 960-1295 Japan; 3 Department of Pediatrics, Fukushima Medical University School of Medicine, 1 Hikarigaoka, Fukushima, 960-1295 Japan; 4 Department of Public Health, Fukushima Medical University School of Medicine, 1 Hikarigaoka, Fukushima, 960-1295 Japan; 5 Fukushima Medical Center for Children and Women, Fukushima Medical University, 1 Hikarigaoka, Fukushima, 960-1295 Japan

**Keywords:** Preconception dysmenorrhea, Preterm birth, Small-for-gestational-age infant, Preterm premature rupture of membrane, Hypertensive disorders of pregnancy

## Abstract

**Background:**

The association of maternal preconception dysmenorrhea, especially primary dysmenorrhea, with obstetric complications has not been clearly described. Therefore, we evaluated the association of preconception dysmenorrhea with obstetric complications while accounting for the presence of pelvic pathologies.

**Methods:**

We analyzed the data of women with singleton live births at and after 22 weeks of gestation enrolled in the Japan Environment and Children’s Study, a nationwide birth cohort study, between 2011 and 2014. Participants with psychological disorders were excluded. Preconception dysmenorrhea, identified in the medical record transcripts, was categorized into mild dysmenorrhea (MD) and severe dysmenorrhea (SD). Furthermore, excluding those who had pelvic pathologies via self-reported questionnaires (endometriosis, adenomyosis, and uterine myomas) with MD and SD, preconception dysmenorrhea was categorized into mild primary dysmenorrhea (MPD) and severe primary dysmenorrhea (SPD), respectively. Using multiple logistic regression, adjusted odds ratios (aORs) for obstetric complications, including preterm birth (PTB) before 37 weeks and 34 weeks, small-for-gestational-age infants, preterm premature rupture of membrane, and hypertensive disorders of pregnancy, were calculated (considering confounders) in women with (1) MD or SD and (2) MPD or SPD. Women without preconception dysmenorrhea were used as a reference.

**Results:**

A total of 80,242 participants were analyzed. In women with SD, the aOR for PTB before 37 weeks was 1.38 (95% confidence interval [CI] 1.10, 1.72). In women with SPD, the aOR for PTB before 37 weeks was 1.32 (95% CI 1.02, 1.71). There was no association between women with MD or MPD and obstetric complications.

**Conclusions:**

SD and SPD are significantly associated with an increased incidence of PTB before 37 weeks. Care providers should provide proper counseling regarding the association between preconception dysmenorrhea and obstetric complications. Optimal management of pregnant women with preconception dysmenorrhea to reduce the incidence of PTB should be elucidated in further studies, with detailed clinical data of pelvic pathologies.

## Background

Menstrual disorders, including dysmenorrhea and irregular periods, are important indicators of hormonal imbalance, inflammation, and risk of future health issues in women [1–3]. Dysmenorrhea, defined as painful menstrual cramps of uterine origin, is a frequent gynecological condition occurring before pregnancy, with a prevalence of 16–95% [2, 3]. According to a survey on menstrual symptoms conducted in 2011 that included approximately 20,000 Japanese women aged 15–49 years, about half of the participants reported pain as a menstrual symptom [4]. Dysmenorrhea is subclassified into either primary or secondary dysmenorrhea, depending on the presence of discernible macroscopic pelvic pathologies such as endometriosis, adenomyosis, and uterine myomas [5]. These pelvic pathologies have been reported to affect several obstetric outcomes [6–12].

Obstetric complications, such as preterm birth (PTB), small-for-gestational-age (SGA) infants, and representative causative factors of PTB, such as preterm premature rupture of membrane (pPROM) and hypertensive disorders of pregnancy (HDP), are potential leading causes of neonatal mortality and morbidity. Rowlands et al. [13] showed that severe period pain and heavy menstrual periods were associated with PTB in a recent population-based cohort study. Juang et al. [14] reported that severe primary dysmenorrhea (SPD) was associated with an increased risk of spontaneous PTB in a case-control study including 329 singleton PTB cases and 329 singleton gravid women with term delivery as controls. Similarly, several studies have reported the association between preconception dysmenorrhea and obstetric complications, such as low-birth-weight infants [15], pPROM [14], HDP [16], and psychological distress during pregnancy [17]. However, there remains a dearth of research evaluating the association between preconception dysmenorrhea (especially primary dysmenorrhea, i.e., dysmenorrhea in the absence of pelvic pathologies) and obstetric complications using data from large study populations.

We hypothesized that preconception dysmenorrhea, including primary dysmenorrhea, is associated with a higher incidence of obstetric complications than that in the general population, according to previous studies. Therefore, in this study, we aimed to clarify the association of preconception dysmenorrhea and preconception primary dysmenorrhea with PTB, SGA infant, pPROM, and HDP, using data from a nationwide Japanese birth cohort study.

## Methods

### Study design

We analyzed the data from the Japan Environment and Children’s Study (JECS), which is a nationwide, government-funded, prospective birth cohort study that started in January 2011, to investigate the effects of environmental factors on children’s health [18, 19]. Briefly, the JECS was funded directly by the Ministry of the Environment, Japan, and involved collaboration among the Programme Office (National Institute for Environmental Studies), Medical Support Centre (National Center for Child Health and Development), and 15 Regional Centres (Hokkaido, Miyagi, Fukushima, Chiba, Kanagawa, Koshin, Toyama, Aichi, Kyoto, Osaka, Hyogo, Tottori, Kochi, Fukuoka, and South Kyushu/Okinawa) [18, 19]. For inclusion in the JECS, expectant mothers had to meet the following criteria: (1) residence within the Study Area at the time of recruitment and expected to continue residing in Japan in the foreseeable future; (2) expected due date between August 1, 2011, and mid-2014; and (3) cognitive and Japanese language abilities required to participate in the JECS to complete a self-administered questionnaire. The JECS protocol was reviewed and approved by the Ministry of the Environment Institutional Review Board on Epidemiological Studies (No. 100910001) and by the Ethics Committees of all participating institutions. The JECS was conducted in accordance with the Helsinki Declaration and other national regulations and guidelines. Written informed consent was obtained from all participants.

There were two modes of recruitment: (1) during the first prenatal examination by cooperating health-care providers and (2) at local government offices that issued a pregnancy journal, called the Maternal and Child Health Handbook, to all expecting mothers in Japan before they received municipal services for pregnancy, delivery, and childcare. Pregnant women were contacted by cooperating health-care providers or local government offices issuing the Maternal and Child Health Handbooks, and those willing to participate were registered. Data on demographic factors, medical history, physical and mental health, lifestyle, occupation, environmental exposures at home and in the workplace, housing conditions, and socioeconomic status were collected from the responses in the self-administered questionnaires to the participating expectant mothers [18, 19].

### Data collection

The current analysis used the data released in October 2019 (data set: jecs-ta-20190930). Specifically, we used three types of data: (1) M-T1, comprising data on maternal medical background factors, obtained through a self-reported questionnaire during the first trimester (first questionnaire); (2) M-T2, comprising data on partner lifestyle and socioeconomic status, obtained through a self-reported questionnaire during the second or third trimester (second questionnaire); and (3) Dr-0 m, comprising data on obstetric outcomes, such as gestational age and birthweight, collected throughout pregnancy and extracted from medical record transcripts provided by cooperating health-care providers.

Participants with singleton live births at and after 22 weeks of gestation were included. We excluded those with multiple pregnancies, abortions, stillbirths, and deliveries with missing data. Additionally, we excluded participants with psychological disorders and those with Kessler 6 (K6) scores ≥13 [20], as preconception dysmenorrhea is strongly associated with maternal mental disorders, which frequently induce several obstetric complications [21]. Because all missing data were judged to be independent of the exposure and outcome, we performed a complete case analysis (CCA). No significant differences in general characteristics were observed between the included and excluded participants (data not shown).

### Exposure variables, obstetric outcomes, and confounding factors

Information on preconception dysmenorrhea was collected from medical record transcripts. Preconception dysmenorrhea was categorized according to the severity of the menstrual pain and associated symptoms into mild dysmenorrhea (MD) and severe dysmenorrhea (SD). Attending physicians comprehensively judged the severity of patient-reported preconception dysmenorrhea during an interview (including menstrual history and treatment for menstrual disorders, among others) [17], rather than evaluating the severity of dysmenorrhea using the visual analog scale and numeric scale [22]. Women who could perform daily activities despite the menstrual pain were deemed as having MD, whereas those who needed bed rest due to the menstrual pain were deemed as having SD [17].

Additionally, excluding those who had pelvic pathologies indicated via self-reported questionnaires (endometriosis, adenomyosis, and uterine myomas) with MD and SD, preconception dysmenorrhea was categorized into mild primary dysmenorrhea (MPD) and SPD, respectively. There was no information on pelvic pathologies in the medical record transcripts of the JECS; thus, the definition of primary dysmenorrhea was just based on self-administered questionnaires: “self-reportedly defined primary dysmenorrhea.”

The definition of PTB was births before 37 weeks and 34 weeks of gestation, and SGA infant was defined as an infant with birthweight < 1.5 standard deviations (corrected for parity, gestational age, and sex) on the new Japanese neonatal anthropometric charts for gestational age at birth [23]. The definition of pPROM was a spontaneous rupture of membranes before 37 gestational weeks. The definition of HDP was persistently elevated blood pressure (≥140/90 mmHg) after 20 gestational weeks in an otherwise normotensive woman [24]. These data were retrieved from the medical record transcripts.

The following items were considered as potential confounding factors: maternal age, parity, maternal smoking and alcohol consumption status, maternal educational status, annual household income, and maternal pre-pregnancy body mass index (BMI) and gestational weight gain (GWG). Maternal age was categorized into three groups (< 20, 20–34, and ≥ 35 years) based on a previous study reporting that maternal age was associated with certain obstetric complications, such as PTB and SGA infants [25, 26]. Parity was categorized into two groups (nulliparous and multiparous). The smoking status of the participants was retrieved from the selection of the following options in the questionnaire: “Currently smoking,” “Never,” “Previously did, but quit before realizing current pregnancy,” and “Previously did, but quit after realizing current pregnancy.” The smoking category included the maternal participants who chose “Currently smoking,” whereas the non-smoking category consisted of the remaining participants. Similarly, the information on participants’ alcohol consumption status was retrieved by their choices among the following: “never drank,” “quit drinking before pregnancy,” “quit drinking during early pregnancy,” and “kept drinking during pregnancy” [27]. The drinking category included participants who chose “kept drinking during pregnancy;” all other participants were included in the non-drinking category. Maternal educational status was categorized into four groups based on the completed number of years of education (junior high school, < 10 years; high school, 10–12 years; technical/vocational college or university, 13–16 years; and graduate school, ≥17 years). Annual household income was categorized into four levels (< 2,000,000, 2,000,000–5,999,999, 6,000,000–9,999,999, and ≥ 10,000,000 JPY). Maternal pre-pregnancy BMI was categorized as < 18.5, 18.5–24.9, and ≥ 25.0 kg/m^2^ into three groups [28]. GWG was calculated as the difference in the bodyweight just before delivery and the bodyweight before pregnancy (kg). We defined appropriate GWG as < 12 kg and excessive GWG as ≥12 kg, according to the criteria defined by the Ministry of Health, Labor and Welfare of Japan [29]. Appropriate GWG was defined as 9–12 kg for women with pre-pregnancy BMI < 18.5 kg/m^2^, and 7–12 kg for women with pre-pregnancy BMI between 18.5 and 24.9 kg/m^2^, and was individually assessed for women with pre-pregnancy BMI ≥ 25.0 kg/m^2^. These confounding factors were selected based on clinical importance.

### Statistical analyses

Participants were stratified based on the presence of preconception dysmenorrhea, and clinical and demographic sample characteristics were compared between groups. The chi-square test was used to compare the characteristics (expressed as categorical variables) between groups. Additionally, multiple logistic regression models were used to calculate crude odds ratios (cORs), adjusted odds ratios (aORs), and 95% confidence intervals (CIs) for PTB before 37 weeks and 34 weeks, SGA infant, pPROM, and HDP in women with (1) MD or SD, and (2) MPD or SPD, using those without preconception dysmenorrhea as a reference, after controlling simultaneously for the following potential confounders: maternal age, parity, maternal smoking and alcohol consumption status, maternal educational status, annual household income, maternal pre-pregnancy BMI, and excessive GWG. SPSS version 26 (IBM Corp., Armonk, NY, USA) was used for statistical analyses. A *P* value < 0.05 indicated statistical significance.

## Results

**Fig. 1:**
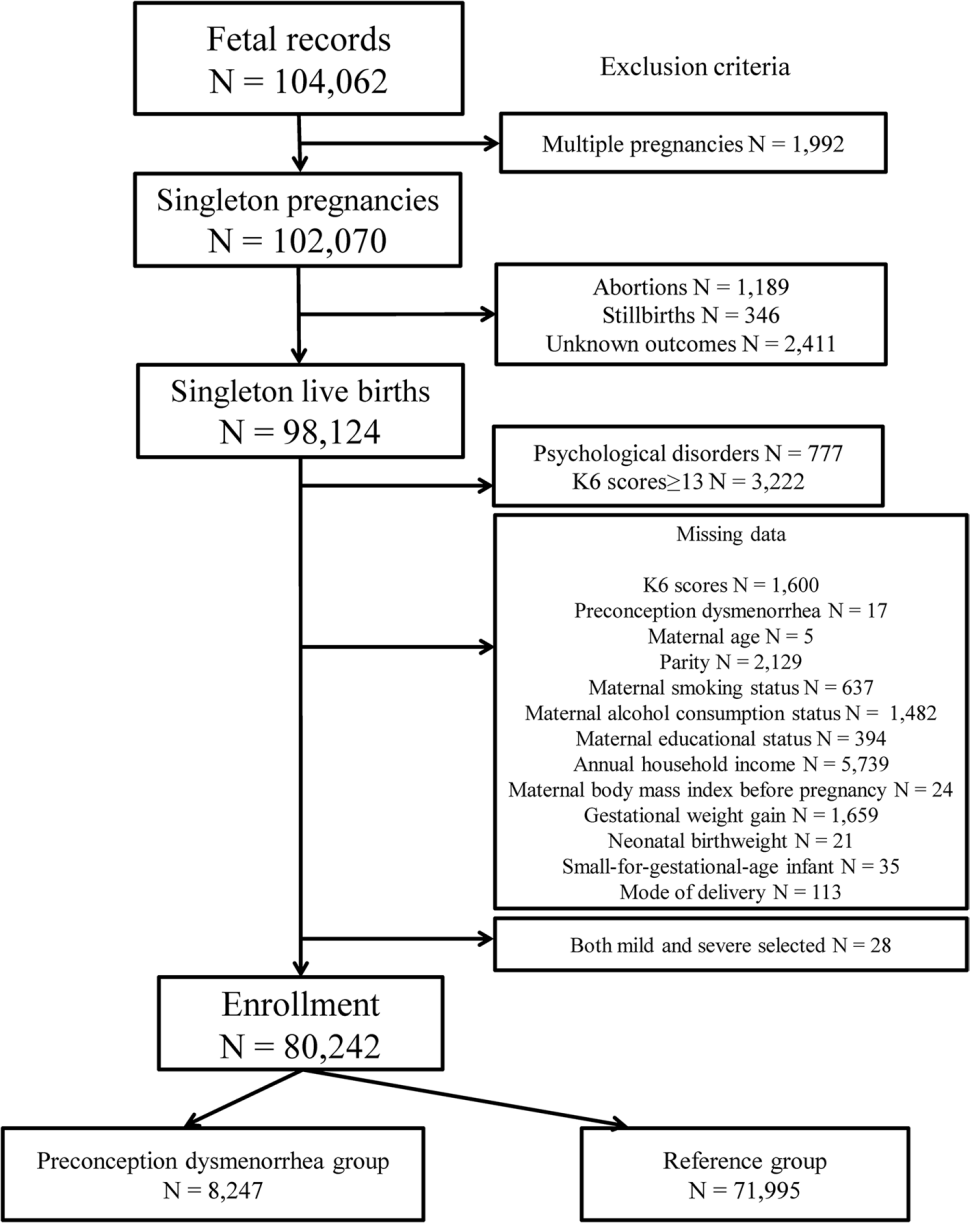
Flowchart depicting participant eligibility for study enrolment. Abbreviation: K6, Kessler 6 score

The total number of fetal records in the JECS was 104,102. A total of 80,242 participants met the inclusion criteria (Fig. 1), excluding those with multiple pregnancies in 1992 cases, abortions in 1189 cases, stillbirths in 346 cases, and deliveries with unknown outcomes in 2411 cases. Additionally, we excluded participants with psychological disorders in 777 cases and those with K6 scores ≥13 for 3222 cases. There were missing data regarding K6 scores for 1600 cases, preconception dysmenorrhea for 17 cases, maternal age for 5 cases, parity for 2129, maternal smoking status for 637, maternal alcohol consumption status for 1482, maternal educational status for 394, annual household income for 5739, maternal pre-pregnancy BMI in 24, GWG for 1659, neonatal birth weight for 21, SGA infants for 35, mode of delivery for 113, and inaccurate dysmenorrhea information for 28. Among the 80,242 included participants, 8247 had preconception dysmenorrhea (preconception dysmenorrhea group), 6784 participants had MD (MD group), 5990 participants had MPD (MPD group), 1463 participants had SD (SD group), and 1163 participants had SPD (SPD group). The remaining 71,995 participants did not have preconception dysmenorrhea (reference group).

Table 1 summarizes the maternal medical background factors and obstetric complications according to the preconception dysmenorrhea status. Significant differences in the distribution of maternal age, maternal educational status, annual household income, maternal pre-pregnancy BMI, and ratios of nulliparous were observed.

**Table 1:** Maternal medical background factors and obstetric complications according to preconception dysmenorrhea status

	All participants	Preconception dysmenorrhea group	Reference group	*P* value
Variable	*N* = 80,242	*N* = 8247	*N* = 71,995	
Maternal medical background				
Maternal age, % (N)				< 0.001
< 20 years	0.6 (452)	0.7 (58)	0.5 (394)	
20–29 years	71.5 (57,371)	74.3 (6127)	71.2 (51,244)	
≥30 years	27.9 (22,419)	25.0 (2062)	28.3 (20,357)	
Nulliparous, % (N)	39.4 (31,621)	49.0 (4039)	38.3 (27,582)	< 0.001
Smoking during pregnancy, % (N)	4.4 (3567)	4.4 (364)	4.4 (3203)	0.883
Alcohol consumption, % (N)	2.8 (2284)	2.7 (221)	2.9 (2063)	0.337
Maternal education, % (N)				< 0.001
< 10 years	4.3 (3457)	4.4 (366)	4.3 (3091)	
10–12 years	30.7 (24,598)	26.9 (2219)	31.1 (22,379)	
13–16 years	63.6 (50,997)	66.9 (5521)	63.2 (45,476)	
≥17 years	1.5 (1190)	1.7 (141)	1.5 (1049)	
Annual household income, % (N)				0.003
< 2,000,000 JPY	5.5 (4375)	5.1 (419)	5.5 (3956)	
2,000,000–5,999,999 JPY	67.5 (54,172)	66.4 (5474)	67.6 (48,698)	
6,000,000–9,999,999 JPY	22.7 (18,248)	24.3 (2004)	22.6 (16,244)	
≥10,000,000 JPY	4.3 (3447)	4.2 (350)	4.3 (3097)	
Maternal pre-pregnancy BMI, % (N)				< 0.001
< 18.5 kg/m^2^	15.7 (12,629)	18.0 (1482)	15.5 (11,147)	
18.5–25.0 kg/m^2^	73.6 (59,089)	72.2 (5957)	73.8 (53,132)	
≥25.0 kg/m^2^	10.6 (8524)	9.8 (808)	10.7 (7716)	
Excessive GWG (≥ 12 kg), % (N)	29.9 (23,976)	29.9 (2462)	29.9 (21,514)	0.956
Obstetric outcomes				
PTB before 37 weeks, % (N)	4.4 (3507)	4.6 (376)	4.3 (3131)	0.376
PTB before 34 weeks, % (N)	0.8 (670)	0.8 (69)	0.8 (601)	0.986
SGA, % (N)	5.0 (3977)	4.8 (397)	5.0 (3580)	0.529
pPROM, % (N)	1.1 (858)	1.1 (93)	1.1 (765)	0.586
HDP, % (N)	3.1 (2465)	2.9 (236)	3.1 (2229)	0.243

*Abbreviations*: *PTB* preterm birth, *SGA* small-for-gestational-age infant, *HDP* hypertensive disorders of pregnancy, *BMI* body mass index, *GWG* gestational weight gain, *pPROM* preterm premature rupture of membrane

Table 2 summarizes the cORs and aORs for obstetric complications in the MD and SD groups. In the SD group, the cOR and aOR for PTB before 37 weeks were significantly increased relative to those in the reference group (1.34 [95% CI, 1.07–1.68] and 1.38 [95% CI, 1.10–1.72], respectively). In contrast, the aORs for obstetric complications were not increased in the MD group.

**Table 2:** Odds ratios for obstetric complications in women with preconception dysmenorrhea

Obstetric outcomes	PTB < 37 weeks	PTB < 34 weeks	SGA	pPROM	HDP
	Odds ratios (95% CI)				
Exposure					
Reference group	Ref				
MD group *N* = 6784	*N* = 292 (4.3%)	*N* = 56 (0.8%)	*N* = 332 (4.9%)	*N* = 79 (1.2%)	*N* = 194 (2.9%)
cOR	0.99 (0.88, 1.12)	0.99 (0.75, 1.30)	0.98 (0.88, 1.10)	1.10 (0.87, 1.39)	0.92 (0.79, 1.07)
aOR	1.00 (0.88, 1.13)	0.99 (0.75, 1.30)	0.96 (0.85, 1.07)	1.07 (0.85, 1.35)	0.90 (0.78, 1.05)
SD group *N* = 1463	*N* = 84 (5.7%)	*N* = 13 (0.9%)	*N* = 65 (4.4%)	N = 14 (1.0%)	*N* = 42 (2.9%)
cOR	1.34 (1.07, 1.68)	1.07 (0.61, 1.85)	0.89 (0.69, 1.14)	0.90 (0.53, 1.53)	0.93 (0.68, 1.26)
aOR	1.37 (1.10, 1.72)	1.07 (0.62, 1.87)	0.86 (0.67, 1.10)	0.86 (0.50, 1.46)	0.83 (0.61, 1.14)

*Abbreviations*: *PTB* preterm birth, *SGA* small-for-gestational-age infant, *HDP* hypertensive disorders of pregnancy, *cOR* crude odds ratio, *aOR* adjusted odds ratio, *CI* confidence interval, *pPROM* preterm premature rupture of membrane, *MD* mild dysmenorrhea, *SD* severe dysmenorrhea

Maternal age, parity, maternal smoking status, maternal alcohol consumption status, maternal educational level, annual household income, maternal pre-pregnancy body mass index, and excessive gestational weight gain were used as confounding factors

Table 3 summarizes the cORs and aORs for obstetric complications in the MPD and SPD groups. In the SPD group, the aOR for PTB before 37 weeks was significantly increased relative to that in the reference group (1.32 [95% CI, 1.02–1.71]). In contrast, the aORs for obstetric complications were not increased in the MPD group.

**Table 3:** Odds ratios for obstetric complications in women with preconception primary dysmenorrhea

Obstetric outcomes	PTB < 37 weeks	PTB < 34 weeks	SGA	pPROM	HDP
	Odds ratios (95% CI)				
Exposure					
Reference group	Ref				
MPD group *N* = 5990	*N* = 244 (4.1%)	*N* = 48 (0.8%)	*N* = 286 (4.8%)	*N* = 66 (1.1%)	*N* = 167 (2.8%)
cOR	0.93 (0.82, 1.07)	0.96 (0.71, 1.29)	0.96 (0.85, 1.08)	1.04 (0.81, 1.34)	0.90 (0.77, 1.05)
aOR	0.95 (0.83, 1.08)	0.97 (0.72, 1.31)	0.94 (0.83, 1.06)	1.02 (0.79, 1.32)	0.91 (0.77, 1.07)
SPD group *N* = 1163	*N* = 63 (5.4%)	*N* = 9 (0.8%)	*N* = 53 (4.6%)	N = 9 (0.8%)	*N* = 36 (3.1%)
cOR	1.26 (0.98, 1.63)	0.93 (0.48, 1.79)	0.91 (0.69, 1.20)	0.73 (0.38, 1.41)	1.00 (0.72, 1.40)
aOR	1.32 (1.02, 1.71)	0.96 (0.50, 1.86)	0.88 (0.67, 1.17)	0.70 (0.36, 1.36)	0.95 (0.68, 1.33)

*Abbreviations*: *PTB* preterm birth, *SGA* small-for-gestational-age infant, *HDP* hypertensive disorders of pregnancy, *cOR* crude odds ratio, *aOR* adjusted odds ratio, *CI* confidence interval, *pPROM* preterm premature rupture of membrane, *MPD* mild primary dysmenorrhea, *SPD* severe primary dysmenorrhea

Maternal age, parity, maternal smoking status, maternal alcohol consumption status, maternal educational status, annual household income, maternal pre-pregnancy body mass index, and excessive gestational weight gain were used as confounding factors

## Discussion

This study revealed that SD and SPD were significantly associated with an increased incidence of PTB before 37 weeks; however, no other associations with obstetric complications were observed. Some major strengths of this study include the large sample size, inclusion of data from a nationwide cohort, and robustness of the findings regarding the association of preconception dysmenorrhea with obstetric complications. To the best of our knowledge, this study is the first to clarify the association of primary dysmenorrhea with obstetric complications that provides estimates of the potential risk of obstetric complications using data from a nationwide cohort. The finding of an increased incidence of PTB associated with preconception dysmenorrhea is consistent with that of a previous study on 6615 Australian mothers, in which severe period pain was observed to be associated with an approximately two-fold increase in the incidence of PTB [13]. However, this previous study did not focus on preconception primary dysmenorrhea and used data from self-reported questionnaires only, which was a limitation of the study. Moreover, our findings are partially consistent with those of a previous case-control study involving 658 participants [14], in which the limitation associated with the small study population was strengthened by the large sample size in our study. Thus, in comparison with previous studies, this study has the following strengths: the study population was larger, exposure and outcome measure data were obtained from medical record transcripts, and stratified analyses were conducted based on the presence of pelvic pathologies (self-reportedly defined primary dysmenorrhea).

Although the present findings were based on statistically significant results, careful interpretation regarding clinical implications is warranted. With large sample sizes, the study results should be interpreted carefully to assess whether the significance is clinically meaningful [30]. In this research, the aOR for PTB before 37 weeks in the SD group was 1.37 (95% CI, 1.10–1.72), which can be interpreted to indicate that, at most, the increased risk of PTB is less than double, which could be informative to pregnant women. Moreover, we detected no statistically significant changes in odds ratios for PTB before 34 weeks, in which fetal lung maturation by antenatal corticosteroid is required, and for causal factors of PTB, such as pPROM and HDP. Thus, although careful observation of the pregnancy course in women with preconception dysmenorrhea to prevent PTB by early detection of uterine contractions and cervical changes may be helpful, further studies to clarify the optimal management of pregnant women with preconception dysmenorrhea to reduce the incidence of PTB are needed. Careful interpretation of this study’s results may help pregnant women understand the risks of preconception dysmenorrhea on obstetric complications.

Underlying maternal gynecological conditions in women with preconception dysmenorrhea may play an important role in inducing PTB, as pelvic pathologies frequently cause PTB. Endometriosis and adenomyosis, which are common causes of dysmenorrhea, hypermenorrhea, and irregular periods [5], have been reported to be associated with PTB [6, 7, 10–12]. Moreover, uterine myomas, which also cause dysmenorrhea, are associated with PTB [8]. Additionally, SD may reflect a high inflammatory condition, which affects the incidence of PTB. Furthermore, this study revealed the association of SPD with an increased incidence of PTB. Primary dysmenorrhea is a complex chronic condition, and its most widely accepted etiology is the overproduction of uterine prostaglandins, which induces pain and inflammation [31]. Furthermore, vasopressin may be related to the etiology of primary dysmenorrhea, which may result in excessive uterine contractions [32]. The underlying excess of prostaglandins and vasopressin in women with preconception primary dysmenorrhea may cause excessive uterine contractions during pregnancy and lead to PTB. In this study, the mean gestational age at birth among pregnant women with SPD was over 34 gestational weeks (data not shown), which may strengthen the speculation that PTB is caused by hormonal effects rather than by intrauterine infection in this patient group.

This study has some limitations. First, there might have been potential risks associated with exposure misclassification and unmeasured confounding factors in this study. According to a previous study, the prevalence of dysmenorrhea in Japanese women was approximately 50%, which was much higher than that observed in this study. Moreover, there were no unified criteria to classify the severity of preconception dysmenorrhea, compared with evaluating the severity of dysmenorrhea using the visual analog scale and numeric scale [22]. Furthermore, information regarding pre-pregnancy treatments, including pain medication and hormonal treatments as well as the differences in the location and region of the participants were also lacking. Because these factors may influence the association between the severity of preconception dysmenorrhea and the incidence of obstetric complications, our results need to be interpreted cautiously, including the generalizability of the results. Second, the criteria for pelvic pathologies were not standardized, and this information was based on self-reported questionnaires. Therefore, there might have been participants with unknown conditions associated with pelvic pathologies. Further research is needed to clarify the effects of preconception dysmenorrhea on obstetric complications, including clinical information on various pelvic pathologies to confirm the results of this study. Because reliable diagnosis of primary dysmenorrhea is based on ultrasonography to detect pelvic pathologies in all patients with dysmenorrhea, a prospective observational study is needed to truly elucidate this association. Lastly, performing CCA in this study might have led to potentially biased results. Although CCA was judged to be suitable because missing data were jointly independent of the exposure and outcome [33, 34], and there were no significant differences in characteristics between the included and excluded patients, careful interpretation of the results may be needed.

## Conclusions

Both SD and SPD were statistically significantly associated with an increased incidence of PTB before 37 weeks. Clinicians need to consider these data for proper counselling on the association of preconception dysmenorrhea with obstetric complications. Optimal management of pregnant women with preconception dysmenorrhea to reduce the incidence of PTB should be elucidated in further studies, with detailed clinical data on pelvic pathologies.

## Data Availability

Data are unsuitable for public deposition due to ethical restrictions and legal framework of Japan. It is prohibited by the Act on the Protection of Personal Information (Act No. 57 of 30 May 2003, amendment on 9 September 2015) to publicly deposit the data containing personal information. Ethical Guidelines for Epidemiological Research enforced by the Japan Ministry of Education, Culture, Sports, Science and Technology and the Ministry of Health, Labor and Welfare also restricts the open sharing of the epidemiologic data. All inquiries about access to data should be sent to: jecs-en@nies.go.jp. The person responsible for handling enquiries sent to this e-mail address is Dr. Shoji F. Nakayama, JECS Programme Office, National Institute for Environmental Studies.

## Abbreviations

aOR: Adjusted odds ratio
BMI: Body mass index
CCA: Complete case analysis
CI: Confidence interval
cOR: Crude odds ratio
GWG: Gestational weight gain
HDP: Hypertensive disorders of pregnancy
JECS: Japan Environment and Children’s Study
K6 score: Kessler 6 score
MD: Mild dysmenorrhea
MPD: Mild primary dysmenorrhea
pPROM: Preterm premature rupture of membrane
PTB: Preterm birth
SD: Severe dysmenorrhea
SGA: Small-for-gestational-age infant
SPD: Severe primary dysmenorrhea
